# Anthropometry in Children

**Published:** 1899-09

**Authors:** 


### Anthropometry in Children.

It appears to be a law in medicine and in applied science in general that the more the literature of a new field of activity is sub-divided, the slower is the advance, and the smaller is the total of practical utility and of general recognition of excellence on the part of the understanding public. The comparatively new field of physical mensuration and psycho-physical tests is already divided up among a number of zealous students, including anthropologists, physiologists, pedagogues, criminologists, neurologists, alienists, etc., etc. It is certainly worthy of note that the pediatrists, as a class, have been somewhat backward in identifying themselves with a movement that ought to appeal to them closely. In a subject containing the great possibilities of anthropometry, co-operation is imperative to insure success. A federal society should be formed, with annual meetings, transactions, and official organ. Prominent representatives of all scientific walks in which anthropometry is indispensable to progress should identify themselves with this society. Especially should any physician become a member who intends to devote his life to the study of pediatrics. The recently issued Government report, by MacDonald, entitled "Experimental Study of Children," will give the medical man an insight into the possibilities of anthropometry as applied to children. It is extremely desirable to secure measurements and tests of children in any way out of the common. If they are especially bright, or stupid, or of mixed ancestry, or vicious, or unduly docile, or if they offer peculiar mental, moral or physical traits or capabilities, and so through a score of deviations from the average, the careful registration of these peculiarities may ultimately bring about, among numerous other equally important desiderata, the development of a science which will replace, to some extent, the crude phrenologies, physiognomies and other aberrant strivings after certitude in character estimation. From this standpoint, who is so well qualified as the physician to first recognize unusual types of childhood? Cases of this sort reported to the parent society would doubtless find an echo not only from other physicians, but from educators, penologists, artists, etc., etc. MacDonald's book is full of generalizations, and shows how pre-eminently anthropometry is calculated to find expression in laws or axioms. To select a few at random: "Bright boys are in general taller and heavier than dull boys." "Mixture of nationality is unfavorable to mental ability." "Colored children grow brighter with age; white children do not." Let us again urge that the field of this interesting study be no longer split up, and that all scientists who are interested in anthropometry lose no time in getting together and providing a common meeting-ground for the exchange of ideas.—*Pediatrics.*

